# Development of a PacBio Long-Read Sequencing Assay for High Throughput Detection of Fungicide Resistance in *Zymoseptoria tritici*

**DOI:** 10.3389/fmicb.2021.692845

**Published:** 2021-06-18

**Authors:** Berit Samils, Björn Andersson, Eva Edin, Malin Elfstrand, Tilman Rönneburg, Diana Bucur, Fiona Hutton, Thies M. Heick, Pierre Hellin, Steven Kildea

**Affiliations:** ^1^Department of Forest Mycology and Plant Pathology, Swedish University of Agricultural Sciences, Uppsala, Sweden; ^2^Department of Medical Biochemistry and Microbiology, Uppsala University, Uppsala, Sweden; ^3^TEAGASC, The Agriculture and Food Development Authority, Carlow, Ireland; ^4^Department of Agroecology, Aarhus University, Aarhus, Denmark; ^5^Plant and Forest Health Unit, Walloon Agricultural Research Center, Gembloux, Belgium

**Keywords:** azole, SDHI, QoI, multiplex PCR, housekeeping genes, SNP markers, SSR markers

## Abstract

Fungicide resistance has become a challenging problem in management of Septoria tritici blotch (STB), caused by *Zymoseptoria tritici*, the most destructive disease of winter wheat throughout western and northern Europe. To ensure the continued effectiveness of those fungicides currently used, it is essential to monitor the development and spread of such resistance in field populations of the pathogen. Since resistance to the key families of fungicides used for STB control (demethyalation inhibitors or azoles, succinate dehydrogenase inhibitors or SDHIs and Quinone outside Inhibitors or QoIs) is conferred through target-site mutations, the potential exists to monitor resistance through the molecular detection of alterations in the target site genes. As more efficient fungicides were developed and applied, the pathogen has continuously adapted through accumulating multiple target-site alterations. In order to accurately monitor these changes in field populations, it is therefore becoming increasingly important to completely sequence the targeted genes. Here we report the development of a PacBio assay that facilitates the multiplex amplification and long-read sequencing of the target gene(s) for the azole (*CYP51*), SDHI (*Sdh B*, *C*, and *D*), and QoI (cytochrome *b*) fungicides. The assay was developed and optimised using three Irish *Z. tritici* collections established in spring 2017, which capture the range of fungicide resistance present in modern European populations of *Z. tritici*. The sequences obtained through the PacBio assay were validated using traditional Sanger sequencing and *in vitro* sensitivity screenings. To further exploit the long-read and high throughput potential of PacBio sequencing, an additional nine housekeeping genes (*act*, *BTUB*, *cal*, *cyp*, *EF1*, *GAPDH*, *hsp80-1*, *PKC*, *TFC1*) were sequenced and used to provide comprehensive *Z. tritici* strain genotyping.

## Introduction

The application of fungicides for the control of foliar diseases has become an integral component of winter wheat production throughout Northern Europe. Amongst the targeted-diseases, septoria tritici blotch (STB) is the most commonly encountered and often the most economically destructive (Jørgensen et al., 2014; O’Driscoll et al., 2014). Unfortunately, the casual pathogen *Zymoseptoria tritici* has demonstrated a propensity to adapt and overcome the control provided by these fungicides. This is due to the combination of the high plasticity of the pathogen’s genome and the increasingly specific nature of fungicides used for its control (Lucas et al., 2015; McDonald and Mundt, 2016). Over the past two decades, European *Z. tritici* populations have developed varying degrees of resistance to the majority of fungicides available for use in cereals, including those belonging to the Demethylation Inhibitors (DMIs) of which the azoles are the most abundant and relevant, the Quinone outside Inhibitors (QoIs) and Succinate Dehydrogenase Inhibitors (SDHIs) (Torriani et al., 2009; Leroux and Walker, 2011; Dooley et al., 2016a). The development and spread of such resistances have led to varying efficacy levels provided by the different fungicides (Torriani et al., 2009; Jørgensen et al., 2018, 2021; Joynt et al., 2019). For each fungicide group, multiple mechanisms, including target-site alterations, target-site overexpression and increased efflux activity, have been associated with resistance development (Leroux and Walker, 2011; Cools et al., 2012; Cools and Fraaije, 2013; Omrane et al., 2015; Kildea et al., 2019b).

Target-site alterations are the most common and widespread means through which resistance has developed in *Z. tritici* to the azole, QoI and SDHI fungicides. Nevertheless, in each instance, the nature and impact each have on their respective fungicide group can differ. In the case of QoIs, a single alteration in the pathogen’s cytochrome *b* (G143A) confers complete resistance to all commercially available QoI fungicides (Torriani et al., 2009). On the other hand, resistance to azoles has developed through stepwise accumulation of alterations in multiple positions of their target-site 14α-demethylase (*CYP51*), with varying effects on the sensitivity to the different azoles (Leroux and Walker, 2011; Cools and Fraaije, 2013; Huf et al., 2018). For the SDHIs, multiple individual alterations of the succinate dehydrogenase (*Sdh*) subunits B, C, and D have been detected in field strains with contrasting impacts on SDHI sensitivity (Dooley et al., 2016a; Rehfus et al., 2018). Understanding the dynamics of how these alterations are selected in *Z. tritici* populations is a key component in developing strategies that limit their impacts, both from the aspect of control and further spread. Moreover, it is also highly relevant to be able to rapidly detect the emergence of new alterations or combinations of those already in populations.

Traditionally, the detection of fungicide resistance has relied upon *in vitro* sensitivity analysis. These assays involve measuring fungal growth on/in different media (solid or liquid) amended with increasing concentrations of the fungicide of interest and subsequently determining a given concentration required to inhibit growth by a desired amount (e.g., EC50 or effective concentration of a chemical required to reduce growth by 50%), which can then be used to compare between strains or populations. Although both laborious and economically cumbersome, such phenotypic screenings constitute the foundations of resistance monitoring. As increasing knowledge has become available on the development of fungicide resistance, so did the capacity to supplement these monitoring with detailed molecular studies that assess resistance based on changes at the genetic level. Molecular assays have been applied for the detection of fungicide resistance in *Z. tritici* from simple PCR assays, designed to amplify only in the presence of specific alterations and that can be readily applied in the most basic of laboratories (Siah et al., 2010) to more complex high throughput sequencing assays that generate millions of sequence reads per reaction and are often only available in specialised laboratories or institutes (Pieczul and Wąsowska, 2017). The strengths or weaknesses of each assay depend greatly on the study objectives and desired information.

In most instances, these assays are restricted in their capacity to detect changes in the DNA across short sequences of DNA. This hinders their ability to capture the full information on the haplotype of a strain, which might consist of a combination of multiple alterations which together confer increasing resistance in response to the commercialisation of increasingly active molecules (Cools and Fraaije, 2013). Full haplotype determination is increasingly required for resistance monitoring, as highlighted by the complexity of accumulation of *CYP51* alterations (Leroux and Walker, 2011; Kildea et al., 2019b; Garnault et al., 2021). Whilst it is possible to augment these assays to capture multiple alterations (e.g., multiplexing of different dual-labelled probes to capture multiple alterations in a single qPCR assay as described by Hellin et al., 2020) or indeed entire genes greater than 1 kb (e.g., Frey et al., 2018), these are often either limited in their capacity to capture the diversity present or are highly complex and as such difficult to readily implement. The PacBio long-read sequencing methods based on Single-Molecule Real-Time (SMRT) sequencing technology provides a potential opportunity to overcome both these limitations. SMRT sequencing is capable of generating continuous long-reads with an average read-length of 10 kb or more, and thus can be applied to directly examine the entire haplotypes of the fungicide resistance genes. In addition, an error correction method (circular consensus sequencing; CCS) has been established and repeated sequencing of a single-molecule DNA template can result in extremely high accuracy (Wenger et al., 2019).

Here we report the development of a PacBio long-read sequencing assay built on multiplex amplification of target genes to enable the simultaneous detection of alterations across *CYP51*, *Sdh B*, *C*, and *D* subunits and cytochrome *b*, which, as described above, play a role in conferring resistance in *Z. tritici* to the azole, SDHI and QoI fungicides, respectively. The assay has been optimised to be completed in two multiplex PCRs to ensure high throughput, with an associated pipeline to identify non-synonymous mutations. It can handle 96 samples, with increased scalability possible. To further exploit the high read accuracy associated with SMRT sequencing and the depth of sequencing reads generated in a single run, the multiplex amplification and sequencing of nine housekeeping genes (*act*, *BTUB*, *cal*, *cyp*, *EF1*, *GAPDH*, *hsp80-1*, *PKC*, *TFC1*), alongside those associated with fungicide resistance, have been included to provide comprehensive *Z. tritici* strain genotyping. The ability of the assay to be used for strain genotyping and population diversity analysis was validated using a panel of *Z. tritici* isolates collected from Irish fields in 2017, for which data from Sanger sequencing, PCR-RFLP, SSR, and microtitre plate assays were collected.

## Materials and Methods

### Fungal Isolates and DNA Extraction

In spring 2017, winter wheat leaves carrying STB lesions (final leaf 6) were sampled (a single infected leaf collected every 10 m following a W shape across each field) from commercial fields at three different locations in Ireland prior to fungicide applications (Table 1). Fungal isolations (single pycnidia per infected leaf) were performed as described by Dooley et al. (2016a), after which single spore isolates were generated and subsequently stored at −80°C in 30% glycerol (vv^–1^). DNA was extracted separately at both Teagasc and SLU from the 4-day old single spore isolates cultured on PDA using GeneElute^TM^ (Sigma-Aldrich) and E.Z.N.A. SP Plant DNA Mini Kit (Omega Bio-Tek, Doraville, GA, United States), respectively, in accordance with the manufacturers’ instructions. The collection has previously been described by Kildea et al. (2019a).

**TABLE 1:** Number of *Zymoseptoria tritici* isolates included in each assay.

**Isolate collection**	**Location (Coordinates)**	**No. of *Zymoseptoria tritici* isolates^a^**				
		**PacBio sequencing^b^**	**Sanger sequencing^c^**	**PCR-RFLP^d^**	**SSR**	**Fungicide sensitivity^e^**
Cork	51.9476, −8.0610	36	24–28	32	32	24–26
Meath	53.6476, −6.3008	29	23–26	31	24	25–27
Knockbeg	52.8691, −6.9420	31	25–26	32	28	25–26
Total		96	73–79	95	84	76–77

*^*a*^Number of isolates included in each assay (out of those included in the PacBio sequencing).*

*^*b*^PacBio sequencing of all 14 target genes.*

*^*c*^Sanger sequencing of the *CYP51*, and *SdhB*, *C*, and *D* subunits. The number of isolates sequenced varies for the different target genes.*

*^*d*^PCR-RFLP used for the detection of the cytochrome *b* substitution G143A as reported by Kildea et al. (2019a).*

*^*e*^Sensitivity as determined using *in vitro* microtitre plate assays as described by Dooley et al. (2016b). The number of isolates tested varies for the different fungicides (epoxiconazole, metconazole, prothioconazole-desthio, tebuconazole, benzovindiflupyr, bixafen, boscalid, fluxapyroxad, isopyrazam and penthiopyrad).*

### Development of the PacBio Sequencing Assay

**FIGURE 1:**
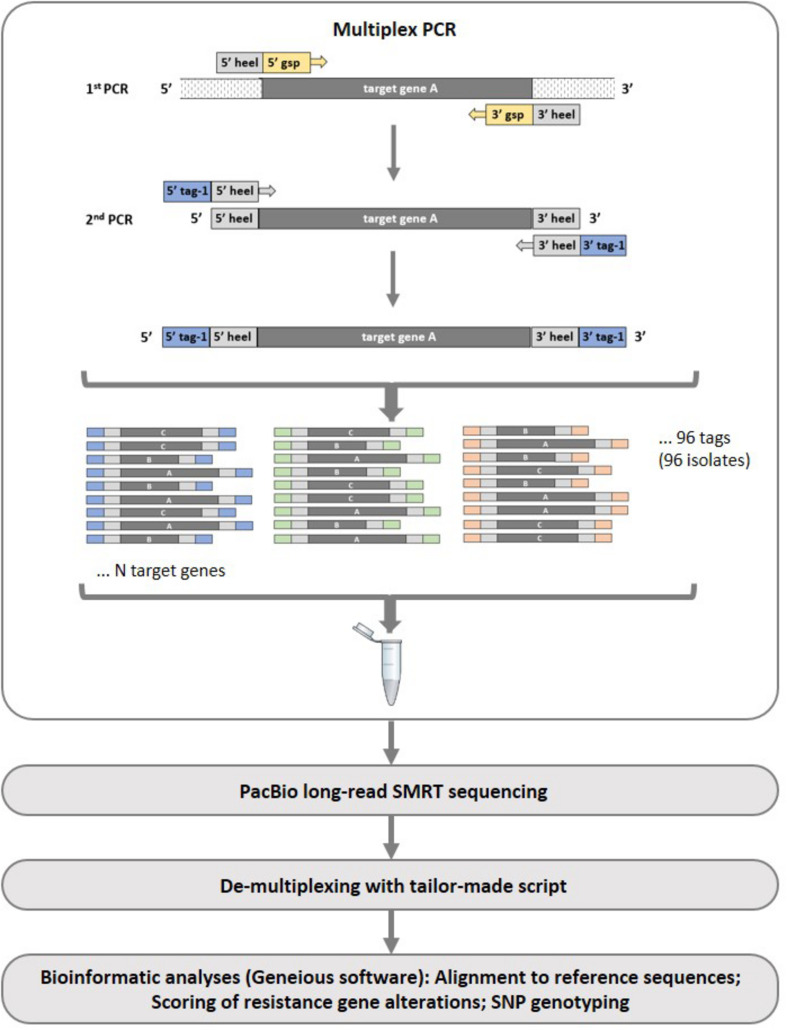
Illustration of the multiplex PCR principal and workflow applied in the PacBio long-read sequencing assay development. In the first multiplex PCR reaction, a number of gene-specific primers (gsp) with heel sequences are used. In the second multiplex PCR reaction, unique tags (one for each reaction tube) are added and used as indexing primers. For simplicity, only one multiplex PCR is shown in the figure, however, amplifications were divided into two PCRs (“A” and “B”) in the current study.

The sequencing assay followed a four-step workflow (Figure 1): (i) amplification of the target genes in individual *Z. tritici* isolates using multiplex PCRs including indexing to allow pooling of isolates, (ii) PacBio long-read sequencing, (iii) de-multiplexing of sequencing reads and assigning consensus sequences to isolates, and (iv) alignment of sequencing reads to reference sequences and mutation/SNP scoring using Geneious Prime software.

#### Multiplex Amplification of Fungicide Resistance and Housekeeping Genes

Primers used for the amplification of the azole (*CYP51*), SDHI (*Sdh* subunits *B*, *C*, and *D*), and QoI (cytochrome *b*) target genes in *Z. tritici* and the nine housekeeping genes obtained from relevant literature or designed in the present study (Table 2). The lengths of amplicons generated for the housekeeping genes were 594–750 bp and 528–2,192 bp for the fungicide target genes. Universal heel sequences, allowing the addition of specific tags (indexing primers), were added to each forward (ctctctatgggcagtc) and reverse primer (ctcgtgtctccgact; Table 2). PCR conditions used for amplification were modified from Chaudhary et al. (2020), originally based on a protocol from Nguyen-Dumont et al. (2013). Due to the varying amplicon sizes, two separate multiplex PCRs were used; PCR “A” for long amplicons (1,200–2,200 bp) and PCR “B” for short amplicons (500–1,000 bp), with extension times and primer concentrations, adapted to amplify all DNA fragments in satisfactory amounts.

**TABLE 2:** Primers used in two multiplex PCRs to amplify fungicide target and housekeeping genes in *Zymoseptoria tritici*.

**Target gene**	**Primer ID**	**Primer sequence (5′-3′) with heel^a^ (at 5′)**	**Amplicon length (bp)**	**PCR**
**Fungicide resistance genes:**				
*CYP51*	CYP51-F1^b^	ctctctatgggcagtcGGGGAAGAAGGGTCGACTTT	2194	A
	CYP51-R4^b^	ctcgtgtctccgactACAGGATGTCGTCTGGATAGT		
*SdhB*	SDH2_ST_1F^c^	ctctctatgggcagtcATACCACACAATGGCTCTTCG	1217	A
	SDH2_ST_1R^c^	ctcgtgtctccgactGTCTTCCGTCGATTTCGAGAC		
*SdhC*	Mg-SdhC-F^d^	ctctctatgggcagtcCCAGTAAGAGGTCCGATTATTACC	903	B
	SdhC-R^e^	ctcgtgtctccgactGCACTCCCTTGGGTCCTGAT		
*SdhD*	SDHD_NEW_1FMG^c^	ctctctatgggcagtcCACTCCTCCAAACCGTATCCT	857	B
	SDHD_NEW_1RMG^c^	ctcgtgtctccgactGGCATCATCGTCAAGCAAG		
*cytb*	Cytb-F^e^	ctctctatgggcagtcGCACGTGGGTAGAGGGTTAT	521	B
	Cytb-R^e^	ctcgtgtctccgactCAGGTGGAGTTTGCATAGGG		
**Housekeeping genes:**				
*act*	Act-F^e^	ctctctatgggcagtcACCTCACTCACACCCTCACC	589	B
	Act-R^e^	ctcgtgtctccgactTCTCCGACGTACGAGTCCTT		
*BTUB*	BTUB-F^e^	ctctctatgggcagtcCCAGTGCGTAAGTAGCACCA	647	B
	BTUB-R^e^	ctcgtgtctccgactGTGATCTGGAAACCCTGGAG		
*cal*	Calm-F^e^	ctctctatgggcagtcCAAGGAAGCCTTCTCCCTCT	608	B
	Calm-R^e^	ctcgtgtctccgactCGGCGGACTTTCTACTTCTG		
*cyp*	Cyclo-F^e^	ctctctatgggcagtcCGCACTATGCACAGGAGAGA	644	B
	Cyclo-R^e^	ctcgtgtctccgactGCCGGGTACTCGTGTAGGTA		
*EF1*	EF1-F^e^	ctctctatgggcagtcTTGACCGTTCTCAAGCCTCT	661	B
	EF1-R^e^	ctcgtgtctccgactCGTAGTACTTGGGGGTCTCG		
*GAPDH*	GAPDH-F^e^	ctctctatgggcagtcAGGGTCTCATGACCACCATC	714	B
	GAPDH-R^e^	ctcgtgtctccgactCTACGCTTGTCCATGCTTGA		
*hsp80-1*	Heat-F^e^	ctctctatgggcagtcTGAGGATCTCCCACTCAACC	667	B
	Heat-R^e^	ctcgtgtctccgactCATGATACGCTCCATGTTGG		
*PKC*	PKC-F^e^	ctctctatgggcagtcGATCGTGAAAAGGCCATCAT	750	B
	PKC-R^e^	ctcgtgtctccgactGGCTGGGAGTGTCCAGACTA		
*TFC1*	TFC1-F^e^	ctctctatgggcagtcGAACGTAGGTGCTGCAAACA	660	B
	TFC1-R^e^	ctcgtgtctccgactGGTGGTGCTGACAGGTTTTT		

*^*a*^Heel forward: ctctctatgggcagtc; Heel reverse: ctcgtgtctccgact.*

*^*b*^From Estep et al. (2015).*

*^*c*^From Dubos et al. (2013).*

*^*d*^From Dooley et al. (2016b).*

*^*e*^From this study.*

Amplification conditions for PCR “A” (long amplicons): The PCR mixture (10 μl) consisted of 1X Phusion HF PCR buffer, 8.3 nM of each *CYP51* primer, 6.7 nM of each *SdhB* primer, 0.2 mM of each dNTP, 0.06 U μl^–1^ Phusion Hot Start II High-Fidelity DNA Polymerase and 5 ng of genomic template DNA. PCR was performed using a 2720 Thermal cycler (AB Applied Biosystems, Life Technologies Corporation, Carlsbad, CA, United States). An initial denaturation step at 98°C for 1 min was followed by 10 cycles of denaturation at 98°C for 30 s, annealing at 61°C for 45 s and extension at 72°C for 2 min 30 s, followed by another 15 cycles of denaturation at 98°C for 30 s, annealing at 59°C for 30 s and extension at 72°C for 2 min 30 s, and thereafter a final extension step at 72°C for 5 min. A second PCR reaction was performed directly afterwards in the same reaction tubes, after adding unique forward and reverse tags (2 μM each) (Supplementary Table 1) to each of the 96 reaction tubes. Thermal cycling conditions consisted of an initial denaturation step at 98°C for 1 min was followed by 3 cycles of denaturation at 98°C for 30 s, annealing at 50°C for 30 s and extension at 72°C for 2 min 30 s, followed by another 10 cycles of denaturation at 98°C for 30 s, annealing at 57°C for 30 s and extension at 72°C for 2 min 30 s, and thereafter a final extension step at 72°C for 10 min.

Amplification conditions for PCR “B” (short amplicons): A similar PCR mixture as for PCR “A” was used, with 8.3 nM of each *SdhC* primer, 6.7 nM of each *SdhD* primer, and 5.6 nM of each primer for the remaining 10 genes [*cytb* (cytochrome *b*), *act* (actin), *BTUB* (beta-tubulin-like gene), *cal* (calmodulin), *cyp* (cyclophilin), *EF1* (elongation factor 1-alpha), *GAPDH* (glyceraldehyde-3-phosphate dehydrogenase), *hsp80-1* (heat stress protein 80-1), *PKC* (protein kinase C), *TFC1* (transcription factor class C)]. The cycling conditions were the same as for PCR “A,” except for an extension time of 60 s (instead of 2 min 30 s) in all cycles, both in the first and second PCR.

All PCR products from the 96 wells were pooled (separately for PCR “A” and “B”) and then purified with AMPure XP beads (Beckman Coulter Genomics, Danvers, MA, United States) using a ratio of 1:0.6 (PCR product:ampure solution) to remove DNA fragments shorter than 300 bp. The PCR products were then further purified with E.Z.N.A. Cycle Pure Kit (Omega Bio-Tek, Doraville, GA, United States). The DNA quality and amplicon sizes were analysed using an Agilent 2100 Bioanalyser (Agilent Technologies, Santa Clara, CA, United States). PCR products of long amplicons (PCR “A”) and short amplicons (PCR “B”) were pooled in equal volumes before PacBio sequencing.

#### PacBio Sequencing

Sequencing was performed at SciLifeLab in Uppsala (NGI, Sweden) with the PacBio Sequel platform (Pacific Biosciences, Menlo Park, CA, United States), which utilises the SMRT technology and circular consensus sequencing (CCS) to produce long reads (Eid et al., 2009). The pooled PCR products from the two multiplex PCRs (containing 14 different amplicons from 96 isolates) were sequenced in one SMRT cell.

#### De-Multiplexing

The multiplexed PacBio sequences were de-multiplexed using an in-house developed script (‘‘detag.py’’)^1^ implemented in Python 2.7^2^, previously described by Chaudhary et al. (2020), to sort reads based on their sequence tag and assign them to their sample of origin. Reads of too low quality (described in detail below), failing to match the heel or tag sequencing, or chimeric reads, were discarded. The script was run with the following arguments: unpairedfastq (unpaired fastq files); h5score 0.9 (proportion of matching 5′ heel bases for acceptance); h3score 0.9 (proportion of matching 3′ heel bases for acceptance); filtering 0:400:2200:30:20 (full filtering, done before the program searches for tags in the sequences : minimum length : maximum length : minimum average per-base phred-score a read needs : minimum phred-score a base needs). Input files with tags and corresponding sample ID (tags file), heel sequences (heel file), and primer sequences (gsp file; for statistics) were supplied to the script. In the processing, reads were organised into new fastq files (labelled with sample ID) with heel and tag sequences removed.

#### Alignments of Sequence Reads

The bioinformatic software Geneious Prime 2019.0.4^3^ was used to align sequencing reads and identify single nucleotide polymorphisms (SNPs) and mutations. The output fastq files from the de-multiplexing script were used as input in Geneious Prime. Alignments were performed with the function “Map to reference.” First, all reads were aligned separately, within sequence lists (i.e., within the sample; option “Assemble each sequence list separately”), to reference sequences of the 14 target genes (Supplementary Table 3). All consensus files were then aligned to the 14 reference sequences, resulting in a list for each target gene, with aligned consensus sequences from all samples. For genes with several exons (*CYP51*, *SdhB*, *SdhC*, and *SdhD*) the nucleotide sequences were aligned to each exon’s reference nucleotide sequences. To find non-synoymous mutations in the fungicide resistance genes, the consensus nucleotide sequences were translated to amino acid sequences using the function “Translate.” All variable amino acid positions throughout the genes were identified by visual examination. The data were copied to an Excel spreadsheet and subsequently compared to those identified through Sanger sequencing.

The nucleotide error rate of PacBio sequencing reads were estimated after quality filtering by the de-multiplexing script. For each targeted gene, the nucleotide error rate was calculated among all reads for ten randomly selected isolates, using the function “Find Variations/SNPs.” The number of nucleotides examined ranged from 44,000 to 1,009,000 per gene, and in total 8,932,000 nucleotides.

The nucleotide consensus reads from the alignment in Geneious Prime were used to locate SNP markers in the housekeeping genes. Variable nucleotide positions in the sequences were indicated by the software. In principle, all SNP sites were selected as markers. To reduce linkage effects, an SNP marker within a distance less than 20 bp to a preceding SNP marker was dismissed when the SNP patterns were similar over samples. The marker set was pruned for singleton SNPs. The SNP data were transferred to an Excel spreadsheet.

### Sanger Sequencing of *CYP51*, *SdhB*, *C*, *D*, and Cytochrome *b* Genes

Amplification, Sanger sequencing and analysis of the *CYP51*, *SdhB*, *C*, and *D* subunits of the 96 isolates of the three collections were performed as previously described by Dooley et al. (2016b) and Kildea et al. (2019a), respectively. Due to the low amplification of the *SdhC* subunit in some isolates, the sequencing assay described by Rehfus et al. (2018) was also used. Sequencing was performed by LGC Genomics (Berlin, Germany). Based on the combination of alterations in *CYP51* gene, isolates were assigned to a *CYP51* haplotype following the nomenclature of Huf et al. (2018), with those not previously reported assigned a new haplotype accordingly. The presence of amino acid substitution G143A in the cytochrome *b*, known to confer QoI resistance, was determined using a PCR-RFLP assay as previously reported by Kildea et al. (2019a). Sanger re-sequencing of ambiguous samples were performed by Macrogen Europe (Amsterdam, Netherlands) using primers pairs CYP51-F1/CYP51-R1, CYP51-F3/CYP51-R3, and CYP51-F4/CYP51-R4 for gene *CYP51* (Estep et al., 2015), and primer pairs according to Table 2 for the *SdhB*, *SdhC*, and *SdhD* genes.

### Fungicide Sensitivity Assessment

All *in vitro* fungicide sensitivity assessments were determined using a microtitre plate assay described by Dooley et al. (2016b), with minor modifications depending on the fungicide. In total, the sensitivities of all 96 strains were determined to four azole fungicides (epoxiconazole, metconazole, prothioconazole-desthio, and tebuconazole) and six SDHI fungicides (benzovindiflupyr, bixafen, boscalid, fluxapyroxad, isopyrazam, and penthiopyrad). In all assays, technical grade fungicides were used and purchased from Sigma-Aldrich (St. Louis, MO, United States), except for benzovindiflupyr (kindly provided by Syngenta). Fungicides were initially dissolved in DMSO and subsequently aliquot to their test concentrations in potato dextrose broth. Fungicides were adjusted to test concentrations of 0, 0.04, 0.123, 0.33, 1.1, 3.3, 10, and 30 mg l^–1^, with the exception of prothioconazole-desthio which concentration was adjusted to give test concentrations of 0, 0.01, 0.04, 0.123, 0.33, 1.1, 3.3, 10 mg l^–1^. Microtitre plates were incubated in the darkness at 18°C for 7 days, after which *Z. tritici* growth was measured as light absorbance using a Synergy-HT plate reader and Gen5 microplate software (BioTek Instruments). Sensitivity was expressed as the concentration of fungicide reducing fungal growth by 50% (EC_50_), which was calculated by fitting a logistic curve to the absorbance data generated for each individual isolate using XL_FIT_ (IDBS Inc.). The sensitivity of the collections to the QoI fungicide pyraclostrobin has been previously reported by Kildea et al. (2019a).

Analysis of variance was performed using Tukey’s studentised range test to determine differences between the three locations regarding the sensitivity of their *Z. tritici* population to each of the 10 fungicides. The analysis was conducted in R 4.0.2 using the packages “car,” “agricolae,” and “ggplot2.”

### Population Diversity Using Microsatellite (SSR) Markers

Isolates were genotyped using 13 SSR markers (ST1, ST10, ST11, ST4, ST5, ST9, ST12, STIE7, ST2, ST3B, ST3C, ST6, and ST7) developed by Gautier et al. (2014) and optimised for the ABI3130xl platform by Welch et al. (2018). Allele calling was performed using GeneMapper^®^, version 3.5 (Applied Biosystems).

### Population Genetic Analysis

The population structure of the *Z. tritici* samples was individually determined based on both SNP marker data from the PacBio sequenced housekeeping genes and microsatellite data. To calculate the level of differentiation among populations, analysis of molecular variance (AMOVA) was done with the Microsoft EXCEL add-in GenAlEx version 6.503 (Peakall and Smouse, 2006, 2012).

## Results

### PacBio Sequencing

A single PacBio sequencing run resulted in a total of 375,804 reads with Phred quality score above Q20 (99% base call accuracy), covering a total of 396,117 Mbp. After processing and checking the quality of the reads using the de-multiplexing script, 88,481 reads (23.5%) were accepted. Reads not accepted were discarded due to low minimum quality (41%), missing primer(s) (17%), missing tag(s) (17.2%), chimeric tags (1.3%), too short length (0.2%), and truncation (<0.1%).

Across all isolates, the average number of reads per targeted gene was between 5.6 and 171.6, with a range between 0 and 556 reads (Table 3). A high overall similarity was found among nucleotides in repeated reads of the same gene for the ten randomly selected isolates, with an overall error rate of 2.21 × 10^–4^, ranging from 0.95 × 10^–4^ to 5.02 × 10^–4^ for the different genes, i.e., 1–5 errors per 10,000 nucleotides (Table 3).

**TABLE 3:** Range of reads per amplicon generated by PacBio sequencing for each gene target and associated error rate^a^.

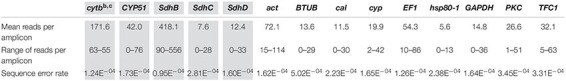

*^*a*^Error rate determined based on comparisons of amplicons generated per individual targets for individual isolates.*

*^*b*^Shaded targets are fungicide specific.*

*^*c*^Full name of gene/protein: *cytb*, cytochrome b, *CYP51*, 14α-demethylase; *SdhB*, succinate dehydrogenase subunit B; *SdhC*, succinate dehydrogenase subunit C; *SdhD*, succinate dehydrogenase subunit D; *act*, actin; *BTUB*, beta-tubulin-like; *cal*, calmodulin; *cyp*, cyclophilin; *EF1*, elongation factor 1-alpha; *GAPDH*, glyceraldehyde-3-phosphate dehydrogenase; *hsp80-1*, heat stress protein 80-1; *PKC*, protein kinase C; *TFC1*, transcription factor class C.*

### Detection of Fungicide Resistance-Associated Alterations and Comparisons With Sanger Sequencing and *in vitro* Sensitivity

#### Cytochrome *b* Sequence and Sensitivity to QoI Fungicides

Using PacBio sequencing, a 521 bp fragment encoding amino acids 96–268 of the cytochrome *b* was successfully amplified and sequenced with a mean read depth of 171.6 reads in each of the 96 isolates (Table 3). The alteration G143A was detected in 92 of the 96 isolates sequenced, which is identical to the detection obtained with the PCR-RFLP assay. No further alterations related to QoI resistance were identified in these isolates or in those without G143A. The sensitivity of 85 of these isolates to the QoI pyraclostrobin was determined with the four isolates with G143 deemed sensitive (EC50 < 0.04 mg l^–1^) and the remaining 81 isolates with G143A ranging from 0.37 to 4.30 mg l^–1^ as previously described by Kildea et al. (2019a).

#### Succinate Dehydrogenase Subunit B, C, and D Sequences and Sensitivity to the SDHI Fungicides

**FIGURE 2:**
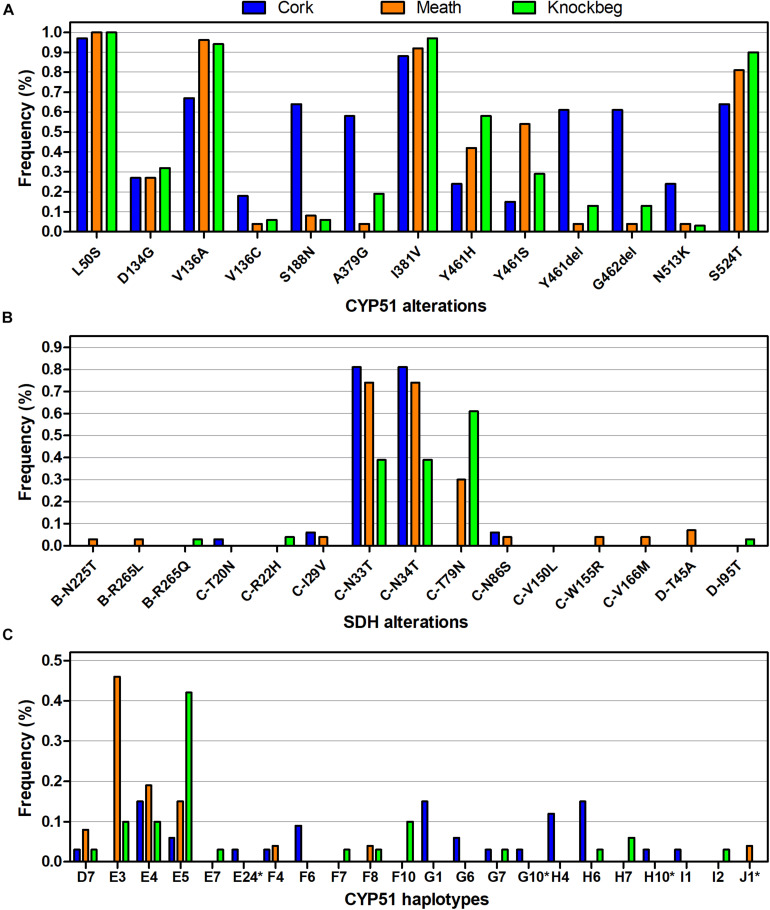
Differences in frequencies of the different **(A)** *SdhB*, *C*, and *D* alterations, **(B)** *CYP51* amino acids, and **(C)** *CYP51* amino acid haplotypes between the three sites sampled. *CYP51* haplotypes as described by Huf et al. (2018), except those marked with asterisk (*), which represents new combinations of amino acid alterations.

**FIGURE 3:**
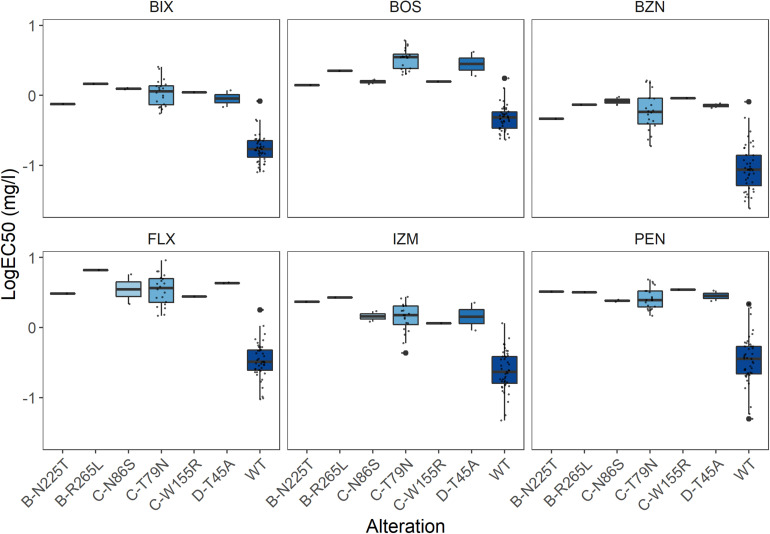
Effect of the different *Sdh* subunit alterations on sensitivity towards the SDHIs bixafen (BIX), boscalid (BOS), benzovindiflupyr (BZN), fluxapyroxad (FLX), izopyrazam (IZM), and penthiopyrad (PEN). Sensitivity presented as LogEC50 (mg l^–1^), with outliers highlighted in bold.

A 1,217 bp sequence covering the entire *SdhB* subunit was successfully obtained for each of the 96 isolates, with a mean read depth of 418.1 reads/isolate (Table 3). Only three non-synonymous mutations were detected amongst the isolates, with the alterations B-N225T identified in two isolates (M16 and C21), and B-R265L and B-R265Q in a single isolate each (M6 and KB31, respectively) (Figure 2A). All three alterations were also identified using Sanger sequencing (a total of 79 isolates compared with both PacBio and Sanger sequencing). Sensitivity for one of the isolates with B-N225T was obtained and resulted in a 3.0–9.8-fold reduction in its sensitivity to the six SDHIs tested compared to those without any *Sdh* subunit alteration. The isolate with B-R265L exhibited a 4.7–19.8-fold reduction in sensitivity (Figure 3). The sensitivity of the isolate with B-R265Q was not determined.

For the *SdhC* subunit, a 903 bp sequence was successfully sequenced in 94 of the 96 isolates tested with a mean read depth of 7.6 reads/isolate. For two isolates (M21 and KB31) no reads were produced. Only a single read was produced for a further seven isolates (M37, C3, C5, C14, C27, KB19, and KB36) and as such, these were not included in the analysis. Amongst the remaining 87 isolates, 10 non-synonymous mutations were identified (Figure 2A). All mutations were confirmed using Sanger sequencing with exception of C-R22H (isolate KB39), for which Sanger sequencing was not performed (a total of 68 isolates compared with both PacBio and Sanger sequencing). As anticipated, only specific alterations, C-T79N, C-N86S, C-V150L & C-V166M, and C-W155R, impacted sensitivity to the different SDHIs, with 3.3–10.5-fold reductions in sensitivity depending on the alteration and SDHI fungicide (Figure 3).

A 857 bp sequence capturing the entire *SdhD* subunit was successfully amplified and sequenced in 92 of the 96 isolates, with a mean read depth of 12.4 reads/isolate. For four isolates (C16, C33, KB5, and M21) no reads were produced. Amongst the 92 isolates successfully sequenced, two non-synonymous mutations were identified (Figure 2A), and their presence was confirmed by Sanger sequencing (a total of 75 isolates compared with both PacBio and Sanger sequencing). In two of the isolates (M23 and M24), this led to the alteration D-T45A, which conferred a 5.2–12.9-fold reduction in sensitivity to the different SDHIs (Figure 3). The alteration D-I95T, found in a single isolate (KB1), did not result in any sensitivity change.

Differences in the frequencies of the various alterations were detected amongst the three sites, with C-T79N most prevalent in Knockbeg and Meath, whilst absent in Cork (Figure 2B). These differences reflected the differing SDHI sensitivities between the three sites (Supplementary Image 1).

#### *CYP51* Sequence and Sensitivity to the Azole Fungicides

A 2,194 bp sequence capturing the entire *CYP51* gene was successfully amplified and sequenced in 92 of the 96 isolates, with a mean read depth of 42 reads/isolate (Table 3). No reads were produced for three isolates (M32, C3, and C5), and the only read for one isolate (M17) was lost due to failure to map to the reference *CYP51* gene. For an additional two isolates (M30 and C26), only a single read was produced, and whilst each mapped to the reference, they were not included in further analysis. Comparison of the translated sequences generated using PacBio sequencing with those generated using Sanger (*n* = 69) showed that the amino acid sequences were identical.

**FIGURE 4:**
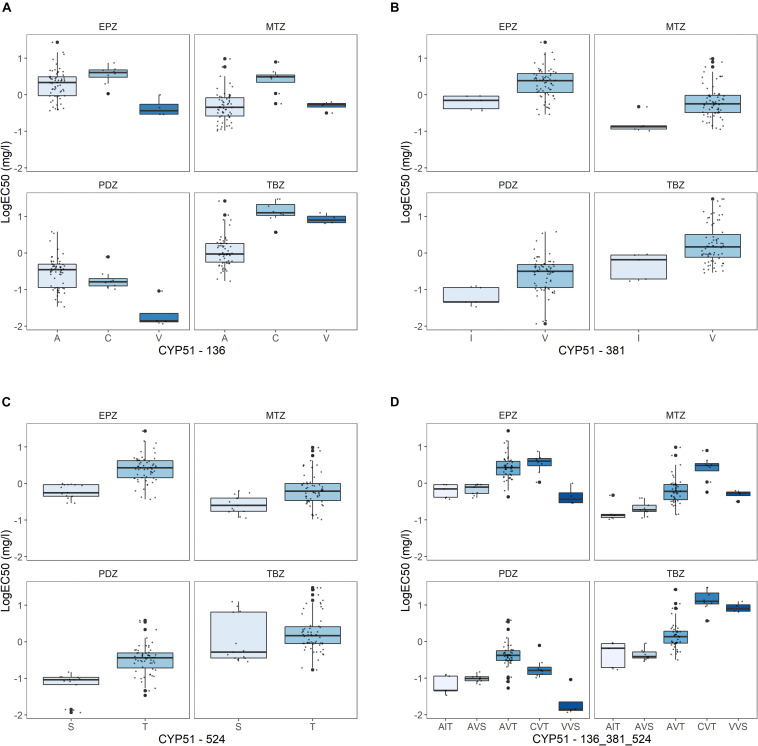
Effect of alterations in the *CYP51* at position **(A)** 136, **(B)** 381, **(C)** 524, and **(D)** at all three positions (the specific amino acid at 136, 381, and 524 in sequential order) on sensitivity towards epoxiconazole (EPZ), metconazole (MTZ), prothioconazole-desthio (PDZ), and tebuconazole (TBZ). Sensitivity presented as LogEC50 (mg l^–1^), with outliers highlighted in bold.

Amongst the 90 isolates successfully sequenced using PacBio, 11 non-synonymous mutations and a 6-bp deletions were detected, resulting in 22 different *CYP51* amino acid haplotypes (Figure 2C). All amino acid alterations detected have previously been reported in *Z. tritici* (Cools and Fraaije, 2013). The frequencies of both *CYP51* amino acid alterations and haplotypes differed between the three sites (Figures 2B,C), reflected in differing levels of sensitivity between the sites to the four azoles tested (Supplementary Image 2). The sensitivity profile of the isolates to the different azoles varied depending on the specific alteration present at *CYP51* amino acid positions 136 (Figure 4A), 381 (Figure 4B), and 524 (Figure 4C). The sensitivity profiles to combinations of alterations at the three amino acid positions are shown in Figure 4D.

### Population Structure Based on Housekeeping Gene Markers and SSR Markers

In total 105 polymorphic SNP markers were identified in the nine housekeeping genes, with 3–25 markers per gene (Supplementary Table 2). The housekeeping gene markers revealed 92 unique multilocus genotypes (MLGs) among the 96 isolates, with 4 MLG’s found twice.

The 13 SSR markers could identify 1–9 alleles per marker, and totally 56 alleles in the entire dataset. The SSR markers distinguished 45 unique MLGs, of which 3 MLGs were found twice, 4 three times, 2 four times, 1 nine times, and 1 was observed fifteen times.

The AMOVA showed that 100% of the variation for the SNP marker data (housekeeping genes) and 97% of the variation for the SSR marker data were found within populations, and no significant differentiation among the three locations was indicated (Table 4).

**TABLE 4:** Analysis of Molecular Variance (AMOVA) for the three *Zymoseptoria tritici* populations using SSR markers and housekeeping gene markers.

	**Source of variation**	**d.f.**	**Sum of squares**	**Variance components**	**Percentage of variation**	***p*-values^a^**
SSR markers	Among populations	2	23,510	194.5	3	0.124
	Within populations	81	513,945	6345.0	97	
Housekeeping gene markers (SNPs)	Among populations	2	19.8	0.0	0	0.67
	Within populations	93	1080.7	11.6	100	

*^*a*^Levels of significance based on 999 permutations.*

## Discussion

With increasing restrictions surrounding the registration and usage of pesticides in the European Union, it is essential to ensure those fungicides that continue to be available and those that are approved into the future remain effective (Jess et al., 2014). Understanding the dynamics of fungicides resistance in target pathogen populations is therefore a key pillar of integrated pest management programmes to control the ensuing diseases. The development of the long-read PacBio assay and associated downstream analysis for detecting target-site fungicide in *Z. tritici*, as detailed here, provide tools to aid such essential research.

To validate the assay, *Z. tritici* populations were collected from three different regions in Ireland collected in spring 2017. They represent a period when the Irish *Z. tritici* population was undergoing considerable changes in terms of fungicide resistance (Dooley et al., 2016a; Kildea et al., 2019b) and, therefore, ideal for testing the robustness of the assay. Based on the phenotypic sensitivity assays varying levels of resistance were present in the collections. Sanger sequencing and a PCR-RFLP assay confirmed the presence of multiple alterations previously identified as conferring azole, SDHI and QoI resistance in their respective target-sites. In all instances, the PacBio assay detected these alterations.

The housekeeping gene markers developed in this study showed high variability and were able to distinguish among multilocus genotypes, even to a greater extent than the SSR markers. The high genotypic diversity indicates that the *Z. tritici* populations are highly recombined as a result of regular cycles of sexual recombination (McDonald and Mundt, 2016). Neither the SSR markers nor the housekeeping gene markers revealed any population structure, i.e., no differentiation between populations and no subgrouping within populations, and the majority of genetic diversity was identified within the fields. The conformity of the two marker sets demonstrates that the housekeeping gene markers, like SSR markers, are neutral and unlinked to the genes involved in fungicide resistance. The lack of population structure among the three Irish fields agree with previous studies, where little geographic differentiation among *Z. tritici* populations was found within Ireland (Welch et al., 2018; Kildea et al., 2019a) as well as on a wider geographic scale (Zhan et al., 2003; McDonald and Mundt, 2016) and is suggested to be explained by high gene flow between populations combined with regular sexual recombination. The differences observed in patterns of fungicide sensitivity and haplotype distribution of fungicide resistance genes among locations, despite the high similarity for neutral markers, suggest that the rate of gene flow may not be sufficient to counteract gene frequency changes caused by ongoing local selection for fungicide resistance. The differences in sensitivity identified between the sites may reflect differences in local fungicide usage. However, it should be noted that the populations examined in this study were collected prior to fungicide application. Even though subtle differences in fungicide usage may occur between the geographic regions from which the samples were obtained, Irish winter wheat fungicide programmes have been broadly similar, relying on combinations of SDHIs and azoles, applied at final leaf three emergence and again at flag leaf emergence. Furthermore, in all instances, the populations were collected from fields that were previously planted to a non-cereal which suggests initial infections would primarily have resulted from airborne ascospore inoculum. As identified by Hellin et al. (2020) *Z. tritici* ascospores are abundant in Ireland in late autumn and early winter and the frequencies of alterations conferring fungicide resistance in these can vary over this period. Further research is required to dissect these differences in fungicide sensitivity.

In the initial design of the assay, a single multiplex PCR assay was trialed; however, due to varying amplicon sizes, larger amplicons were under-represented once sequenced. As a compromise, two initial PCRs were performed with larger fragments (*CYP51* and *SdhB*) amplified together. Nevertheless, as the *SdhB* amplicon is about half the size of the *CYP51*, its average read depth following sequencing was almost 10× greater than for *CYP51* (Table 3). Similar differences were observed for the other amplicons highlighting that further optimisation of the assay should be possible, especially where individual specific targeted amplicon sequencing is required (e.g., *CYP51* only).

The amplification efficiency of individual targeted genes in a multiplex PCR reaction may be impaired in an unpredictable way because of interactions and unequal competition among amplicons for reaction components. For example, shorter amplicons will amplify at a higher rate than longer ones; some primers might be constrained because of primer dimer formations, amplification of certain templates may be favoured due to the properties of the target, the target’s flanking sequences, or differential accessibility of targets within the genome due to secondary structures (Elnifro et al., 2000). In the current assay, individual primer concentrations were adjusted based on empirical testing to promote disadvantaged amplicons. An increase of primer concentrations must, however, be done with caution. The approach of the multiplex PCRs is to keep the concentrations of primers low in the first PCR, ensuring they are depleted before the second PCR, where the added indexing tags will act as primers. If the primers from the first PCR still remain at this stage, they may produce new fragments without tags. Seventeen percent of the reads generated in the present study were rejected in the de-multiplexing step due to lack of tags, which may partly be a result of incomplete depletion of primers in the first PCR.

Depending upon user requirements, the number of targets can be restricted to those relating to fungicide resistance or other specific traits such as those used for genotyping. By reducing the number of targets, the read depth for those remaining will increase. This can be used either to increase the overall number of isolates included or to provide greater depth on those individuals assessed. As the assay is also designed with an initial multiplex PCR, reducing the number of targets in this PCR will also increase the read number of those amplicons under-represented due to the initial PCR process. This potential to increase read depth also opens the assay to be used as a possible quantitative detection method. Phelan et al. (2017) have previously demonstrated the potential of high throughput sequencing to be applied to fungicide resistance detection, with 454-sequencing used to detect the cytochrome *b* alteration G143A in the Irish field populations of the barley pathogen *Rhyncosporium commune*. As identified in the present study, multiple alterations across the entire *CYP51* gene impact the sensitivity to the azole fungicides in a cumulative manner, and similar impacts from multiple *Sdh* subunit mutations were recently reported (FRAC, 2021). Being able to quantify the specific *CYP51* or *Sdh* haplotypes directly from field samples will be invaluable in understanding the dynamics of fungicide resistance following varying treatments.

The fungicide resistant component of the assay described here is specific to target site fungicide resistance. Undoubtedly, this is the predominant form of resistance contributing to erosion in fungicide sensitivity of European *Z. tritici* populations and associated decrease in efficacy observed in the field (Jørgensen et al., 2021). Resistance mechanisms other than target-site alterations, such as target-site overexpression and/or overexpression of efflux pumps, have also been reported in European populations of *Z. tritici* and have the potential to contribute to resistance development (Cools et al., 2012; Omrane et al., 2017). In the case of *CYP51* overexpression conferred by inserts in the promoter region, it should be possible to increase the size of the targeted *CYP51* amplicon to capture some of these key inserts (e.g., 120 bp as described by Cools et al., 2012). Given the increased size of this amplicon, it might be required to amplify it separately as previously highlighted to ensure its adequate representation in the final sequencing. Additional *CYP51* promoter inserts, some of which potentially impacting *CYP51* expressions, have also been identified (Kildea et al., 2019b; Huf et al., 2020). However, as some of these inserts can range in size up to 866 bp, careful amplicon design will be required to ensure the region is effectively captured and sequenced. Given the likely varying fragment sizes that would be amplified in a mixed DNA sample and the resulting bias that may occur, the potential applicability of the assay would be restricted to qualitative detection. This would also be relevant for the detection of inserts in the promoter region of the efflux pump *Mfs1*, which have been associated with increased efflux of a diverse range of fungicides (Omrane et al., 2017).

Although initial setup costs for the PacBio sequencing assay (excluding the costs of the sequencing machines) are slightly more expensive due to the additional PCR step and adapters and indexes, when utilised to its full capacity to screen multiple targets in large collections of isolates, costs per strain can be expected to be considerably lower compared to traditional Sanger sequencing. Future research is ongoing to apply the assay to contemporary collections of *Z. tritici* collected throughout Europe to provide a comprehensive overview of the frequency and spread of fungicide resistance throughout the region.

## Data Availability Statement

The datasets presented in this study can be found in online repositories. The names of the repository/repositories and accession number(s) can be found below: https://www.ncbi.nlm.nih.gov/, PRJNA720680.

## Author Contributions

BA, BS, and SK designed this study and wrote the initial manuscript. ME contributed to the multiplex design. TR created the demultiplexing script. BS, SK, DB, FH, and EE performed the laboratory work and experiments. BS, SK, BA, and DB analysed the data. PH and TH reviewed and edited the manuscript. All authors approved the publication of the manuscript.

## Conflict of Interest

The authors declare that the research was conducted in the absence of any commercial or financial relationships that could be construed as a potential conflict of interest.

## Footnotes

1 http://github.com/troe27/hiplex-detag

2 https://www.python.org

3 https://www.geneious.com

## Supplementary Material

Supplementary Image 1 Differences between the three locations in their sensitivity to the SDHI fungicides; BIX (bixafen), BOS (boscalid), BZN (benzovindifluypr), FLX (fluxopyroxad), IZM (isopyrazam), and PEN (penthiopyrad). Sensitivity presented as LogEC50 (mg l^–1^), with outliers highlighted in bold.

Supplementary Image 2 Differences between the three locations in their sensitivity to the azole fungicides; epoxiconazole (EPZ), metconazole (MTZ), prothioconazole-desthio (PDZ), and tebuconazole (TBZ). Sensitivity presented as LogEC50 (mg l^–1^), with outliers highlighted in bold.

Supplementary Table 1 Indexing tags with heel sequences.

Supplementary Table 2 Summary of SNP markers in housekeeping genes.

Supplementary Table 3 Nucleotide sequences of reference genes in FASTA format.

## References

[B1] Chaudhary R. Rönneburg T. Stein Åslund M. Lundén K. Durling M. B. Ihrmark K. (2020). Marker-trait associations for tolerance to ash dieback in common ash (*Fraxinus excelsior* L.). *Forests* 11:1083. 10.3390/f11101083

[B2] Cools H. J. Bayon C. Atkins S. Lucas J. A. Fraaije B. A. (2012). Overexpression of the sterol 14α-demethylase gene (MgCYP51) in *Mycosphaerella graminicola* isolates confers a novel azole fungicide sensitivity phenotype. *Pest Manag. Sci.* 68 1034–1040. 10.1002/ps.3263 22411894

[B3] Cools H. J. Fraaije B. A. (2013). Update on mechanisms of azole resistance in *Mycosphaerella graminicola* and implications for future control. *Pest Manag. Sci.* 69 150–155. 10.1002/ps.3348 22730104

[B4] Dooley H. Shaw M. W. Mehenni-Ciz J. Spink J. Kildea S. (2016a). Detection of *Zymoseptoria tritici* SDHI-insensitive field isolates carrying the SdhC-H152R and SdhD-R47W substitutions. *Pest Manag. Sci.* 72 2203–2207. 10.1002/ps.4269 26941011

[B5] Dooley H. Shaw M. W. Spink J. Kildea S. (2016b). The effect of succinate dehydrogenase inhibitor/azole mixtures on selection of *Zymoseptoria tritici* isolates with reduced sensitivity. *Pest Manag. Sci.* 72 1150–1159. 10.1002/ps.4093 26269125

[B6] Dubos T. Pasquali M. Pogoda F. Casanova A. Hoffmann L. Beyer M. (2013). Differences between the succinate dehydrogenase sequences of isopyrazam sensitive *Zymoseptoria tritici* and insensitive *Fusarium graminearum* strains. *Pestic. Biochem. Physiol.* 105 28–35. 10.1016/j.pestbp.2012.11.004 24238287

[B7] Eid J. Fehr A. Gray J. Luong K. Lyle J. Otto G. (2009). Real-time DNA sequencing from single polymerase molecules. *Science* 323 133–138. 10.1126/science.1162986 19023044

[B8] Elnifro E. M. Ashshi A. M. Cooper R. J. Klapper P. E. (2000). Multiplex PCR: optimization and application in diagnostic virology. *Clin. Microbiol. Rev.* 3 559–570. 10.1128/CMR.13.4.559-570.2000 PMC88949 11023957

[B9] Estep L. K. Torriani S. F. F. Zala M. Anderson N. P. Flowers M. D. McDonald B. A. (2015). Emergence and early evolution of fungicide resistance in North American populations of *Zymoseptoria tritici*. *Plant. Pathol.* 64 961–971. 10.1111/ppa.12314

[B10] FRAC (2021). *Minutes of the 2021 SDHI Meeting, 20-21^*st*^ January 2021, With Recommendations for 2021.* https://www.frac.info/docs/default-source/working-groups/sdhi-fungicides/sdhi-meeting-minutes/minutes-of-the-2021-sdhi-meeting-20-21th-of-january-2021-with-recommendations-for-2021.pdf?sfvrsn=66eb499a_2 (accessed April 7, 2021).

[B11] Frey R. Widdison S. Scalliet G. Sierotzki H. Walder F. Torriani S. (2018). “Abstracts of presentations at ICPP2018: fungicide sensitivity study of European *Zymoseptoria tritici* populations using large scale phenotyping and targets-based amplicon sequencing,” in *Proceedings of the International Congress of Plant Pathology (ICPP) 2018, 29 July 2018*, (Boston, MA: American Phytopathological Society (APS)), 10.1094/PHYTO-108-10-S1.1

[B12] Garnault M. Duplaix C. Leroux P. Couleaud G. David O. Walker A. S. (2021). Large-scale study validates that regional fungicide applications are major determinants of resistance evolution in the wheat pathogen *Zymoseptoria tritici* in France. *New Phytol.* 229 3508–3521. 10.1111/nph.17107 33226662

[B13] Gautier A. Marcel T. C. Confais J. Crane C. Kema G. Suffert F. (2014). Development of a rapid multiplex SSR genotyping method to study populations of the fungal plant pathogen *Zymoseptoria tritici*. *BMC Res. Notes* 7:373. 10.1186/1756-0500-7-373 24943709 PMC4074386

[B14] Hellin P. Duvivier M. Clinckemaillie A. Charlotte Bataille C. Legrève A. Heick T. M. (2020). Multiplex qPCR assay for simultaneous quantification of CYP51-S524T and SdhC-H152R substitutions in European populations of *Zymoseptoria tritici*. *Plant Pathol.* 69 1666–1677. 10.1111/ppa.132520

[B15] Huf A. Pfleger S. Strobel D. Bryson R. Voegele R. T. Stammler G. (2020). “Distribution and changes of genotypes associated to DMI sensitivity in *Zymoseptoria tritici* in Europe,” in *Modern Fungicides and Antifungal Compounds*, Vol. IX, eds Deising H. B. Fraaije B. Mehl A. Oerke E. C. Sierotzki H. Stammler G. (Braunschweig: DPG Verlag), 93–98.

[B16] Huf A. Rehfus A. Lorenz K. H. Bryson R. Voegele R. T. Stammler G. (2018). Proposal for a new nomenclature for *CYP51* haplotypes in *Zymoseptoria tritici* and analysis of their distribution in Europe. *Plant Pathol.* 67 1706–1712. 10.1111/ppa.12891

[B17] Jess S. Kildea S. Moody A. Rennick G. Murchie A. K. Cooke L. R. (2014). European Union policy on pesticides: implications for agriculture in Ireland. *Pest Manag. Sci.* 70 1646–1654. 10.1002/ps.3801 24753219

[B18] Jørgensen L. N. Hovmoller M. S. Hansen J. G. Lassen P. Clark B. Bayles R. (2014). IPM strategies and their dilemmas including an introduction to www.eurowheat.org. *J. Integr. Agric.* 13 265–281. 10.1016/S2095-3119(13)60646-2

[B19] Jørgensen L. N. Matzen N. Hansen J. Semaskiene R. Korbas M. Danielewicz J. (2018). Four azoles’ profile in the control of *Septoria*, yellow rust and brown rust in wheat across Europe. *Crop Prot.* 105 16–27. 10.1016/j.cropro.2017.10.018

[B20] Jørgensen L. N. Matzen N. Heick T. M. Havis N. Holdgate S. Clark B. (2021). Decreasing azole sensitivity of *Z. tritici* in Europe contributes to reduced and varying field efficacy. *J. Plant Dis. Prot.* 128 287–301. 10.1007/s41348-020-00372-4

[B21] Joynt R. Blake J. Ritchie F. Knight S. Burnett F. Edwards S. (2019). *Fungicide Performance in Wheat, Barley and Oilseed Rape. AHDB Project Report* No. 628. Warwickshire: Agriculture and Horticulture Development Board (AHDB).

[B22] Kildea S. Bucur D. Hutton F. de la Rosa S. Welch T. E. Phelan S. (2019a). Prevalance of QoI resistance and mtDNA diversity in the Irish *Zymoseptoria tritici* population. *Irish J. Agric. Food Res.* 58 27–33. 10.2478/ijafr-2019-0004

[B23] Kildea S. Marten-Heick T. Grant J. Mehenni-Ciz J. Dooley H. (2019b). A combination of target-site alterations, overexpression and enhanced efflux activity contribute to reduced azole sensitivity present in the Irish *Zymoseptoria tritici* population. *Eur. J. Plant Pathol.* 154 529–540. 10.1007/s10658-019-01676-4

[B24] Leroux P. Walker A. S. (2011). Multiple mechanisms account for resistance to sterol 14α-demethylation inhibitors in field isolates of *Mycosphaerella graminicola*. *Pest Manag. Sci.* 67 44–59. 10.1002/ps.2028 20949586

[B25] Lucas J. A. Hawkins N. J. Fraaije B. A. (2015). The evolution of fungicide resistance. *Adv. Appl. Microbiol.* 90 29–92. 10.1016/bs.aambs.2014.09.001 25596029

[B26] McDonald B. A. Mundt C. C. (2016). How knowledge of pathogen population biology informs management of *Septoria tritici* blotch. *Phytopathology* 106 948–955. 10.1094/PHYTO-03-16-0131-RVW 27111799

[B27] Nguyen-Dumont T. Pope B. J. Hammet F. Southey M. C. Park D. J. (2013). A high-plex PCR approach for massively parallel sequencing. *Biotechniques* 55 69–74. 10.2144/000114052 23931594

[B28] O’Driscoll A. Kildea S. Doohan F. Spink J. Mullins E. (2014). The wheat-*Septoria* conflict: a new front opening up? *Trends Plant Sci.* 19 602–610. 10.1016/j.tplants.2014.04.011 24957882

[B29] Omrane S. Audéon C. Ignace A. Duplaix C. Aouini L. Kema G. (2017). Plasticity of the *mfs1* promoter leads to multidrug resistance in the wheat pathogen *Zymoseptoria tritici*. *mSphere* 2:e00393-17. 10.1128/mSphere.00322-18 *mSphere* 3 e00322–18. 29085913 PMC5656749

[B30] Omrane S. Sghyer H. Audéon C. Lanen C. Duplaix C. Walker A. -S. (2015). Fungicide efflux & *MgMFS1* contribute to MDR in *Z. tritici*. *Environ. Microbiol.* 17 2805–2823. 10.1111/1462-2920.12781 25627815

[B31] Peakall R. Smouse P. E. (2006). GENALEX 6: genetic analysis in Excel. Population genetic software for teaching and research. *Mol. Ecol. Notes* 6 288–295. 10.1111/j.1471-8286.2005.01155.x PMC3463245 22820204

[B32] Peakall R. Smouse P. E. (2012). GenAlEx 6.5: genetic analysis in Excel. Population genetic software for teaching and research–an update. *Bioinformatics* 28 2537–2539. 10.1093/bioinformatics/bts460 22820204 PMC3463245

[B33] Phelan S. Barthe M. S. Tobie C. Kildea S. (2017). Detection of the cytochrome *b* mutation G143A in Irish *Rhynchosporium commune* population using targeted 454 sequencing. *Pest Manag. Sci.* 73 1154–1160. 10.1002/ps.4434 27615688

[B34] Pieczul K. Wąsowska A. (2017). The application of next-generation sequencing (NGS) for monitoring of *Zymoseptoria tritici* QoI resistance. *Crop Prot.* 92 143–147. 10.1016/j.cropro.2016.10.026

[B35] Rehfus A. Strobel D. Bryson R. Stammler G. (2018). Mutations in *sdh* genes in field isolates of *Zymoseptoria tritici* and impact on the sensitivity to various succinate dehydrogenase inhibitors. *Plant Pathol.* 67 175–180. 10.1111/ppa.12715

[B36] Siah A. Deweer C. Morand E. Reignault Ph Halama P. (2010). Azoxystrobin resistance of French *Mycosphaerella graminicola* strains assessed by four in vitro assays and by screening of G143A subsituition. *Crop Prot.* 29 737–743. 10.1016/j.cropro.2010.02.012

[B37] Torriani S. F. F. Brunner P. C. McDonald B. A. Sierotzki H. (2009). QoI resistance emerged independently at least 4 times in European populations of *Mycosphaerella graminicola*. *Pest Manag. Sci.* 65 155–162. 10.1002/ps.1662 18833571

[B38] Welch T. Feechan A. Kildea S. (2018). Effect of host resistance on genetic structure of core and accessory chromosomes in Irish *Zymoseptoria tritici* populations. *Eur. J. Plant Pathol.* 150 139–148. 10.1007/s10658-017-1259-9

[B39] Wenger A. M. Peluso P. Rowell W. J. Chang P.-C. Hall R. J. Concepcion G. T. (2019). Accurate circular consensus long-read sequencing improves variant detection and assembly of a human genome. *Nat. Biotechnol.* 37 1155–1162. 10.1038/s41587-019-0217-9 31406327 PMC6776680

[B40] Zhan J. Pettway R. E. McDonald B. A. (2003). The global genetic structure of the wheat pathogen *Mycosphaerella graminicola* is characterized by high nuclear diversity, low mitochondrial diversity, regular recombination, and gene flow. *Fungal Genet. Biol.* 38 286–297. 10.1016/s1087-1845(02)00538-8 12684018

