# Supplementary material for: Development of a PacBio Long-Read Sequencing Assay for High Throughput Detection of Fungicide Resistance in Zymoseptoria tritici
Source: Front Microbiol. 2021 Jun 18;12:692845. doi: 10.3389/fmicb.2021.692845 (PMC8256687; doi:10.3389/fmicb.2021.692845)

Supplementary Figure 1. Differences between the three locations in their sensitivity to the SDHI fungicides; BIX (bixafen), BOS (boscalid), BZN (benzovindifluypr), FLX (fluxopyroxad), IZM (isopyrazam), and PEN (penthiopyrad). Sensitivity presented as LogEC50 (mg l�1), with outliers highlighted in bold.

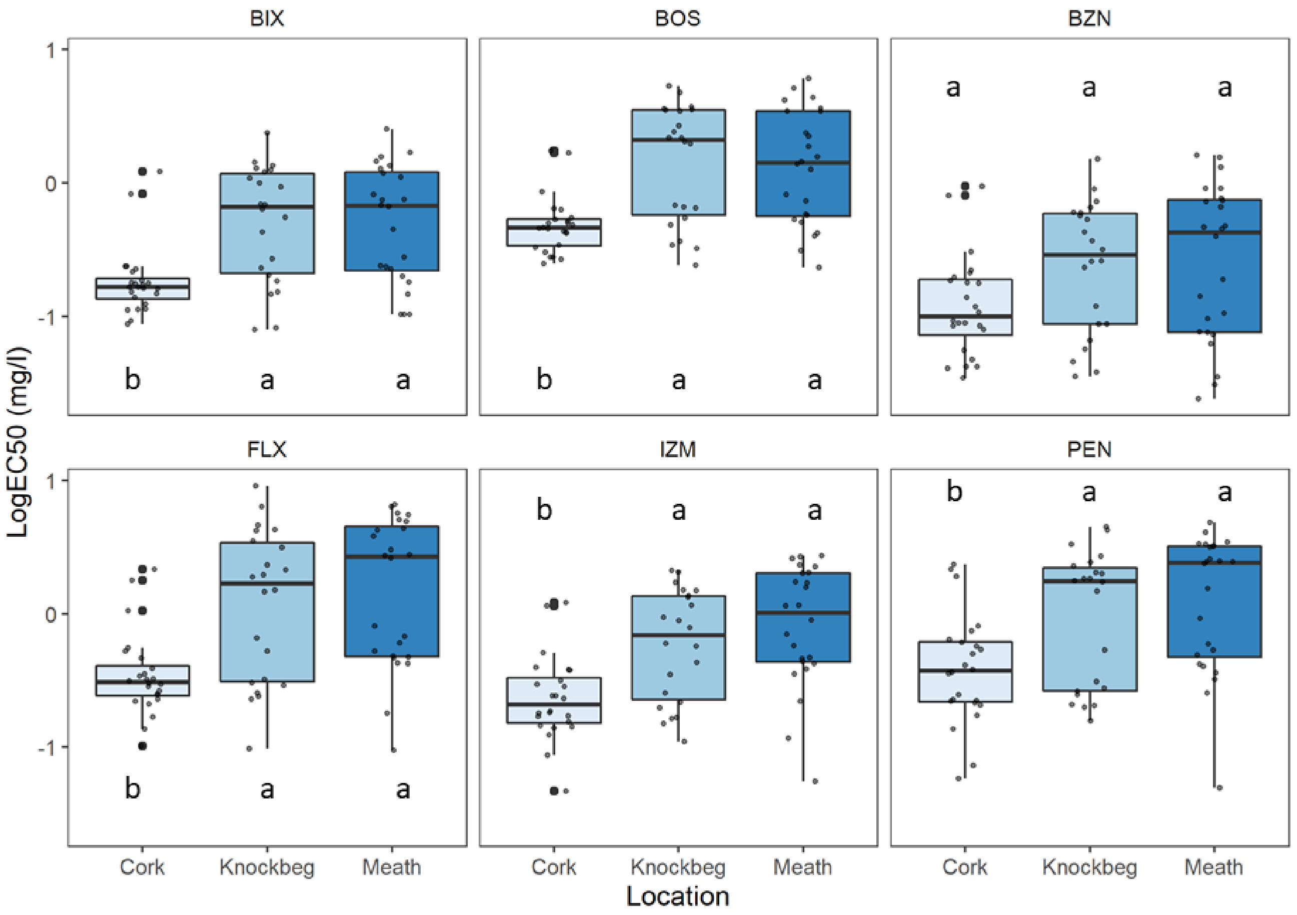

Supplement: Supplementary Image 1 — Differences between the three locations in their sensitivity to the SDHI fungicides; BIX (bixafen), BOS (boscalid), BZN (benzovindifluypr), FLX (fluxopyroxad), IZM (isopyrazam), and PEN (penthiopyrad). Sensitivity presented as LogEC50 (mg l–1), with outliers highlighted in bold. [file Image_1.pdf]
