# Supplementary material for: Development of a PacBio Long-Read Sequencing Assay for High Throughput Detection of Fungicide Resistance in Zymoseptoria tritici
Source: Front Microbiol. 2021 Jun 18;12:692845. doi: 10.3389/fmicb.2021.692845 (PMC8256687; doi:10.3389/fmicb.2021.692845)

Supplementary Figure 2. Differences between the three locations in their sensitivity to the azole fungicides; epoxiconazole (EPZ), metconazole (MTZ), prothioconazole-desthio (PDZ), and tebuconazole (TBZ). Sensitivity presented as LogEC50 (mg l^{-1}), with outliers highlighted in bold.

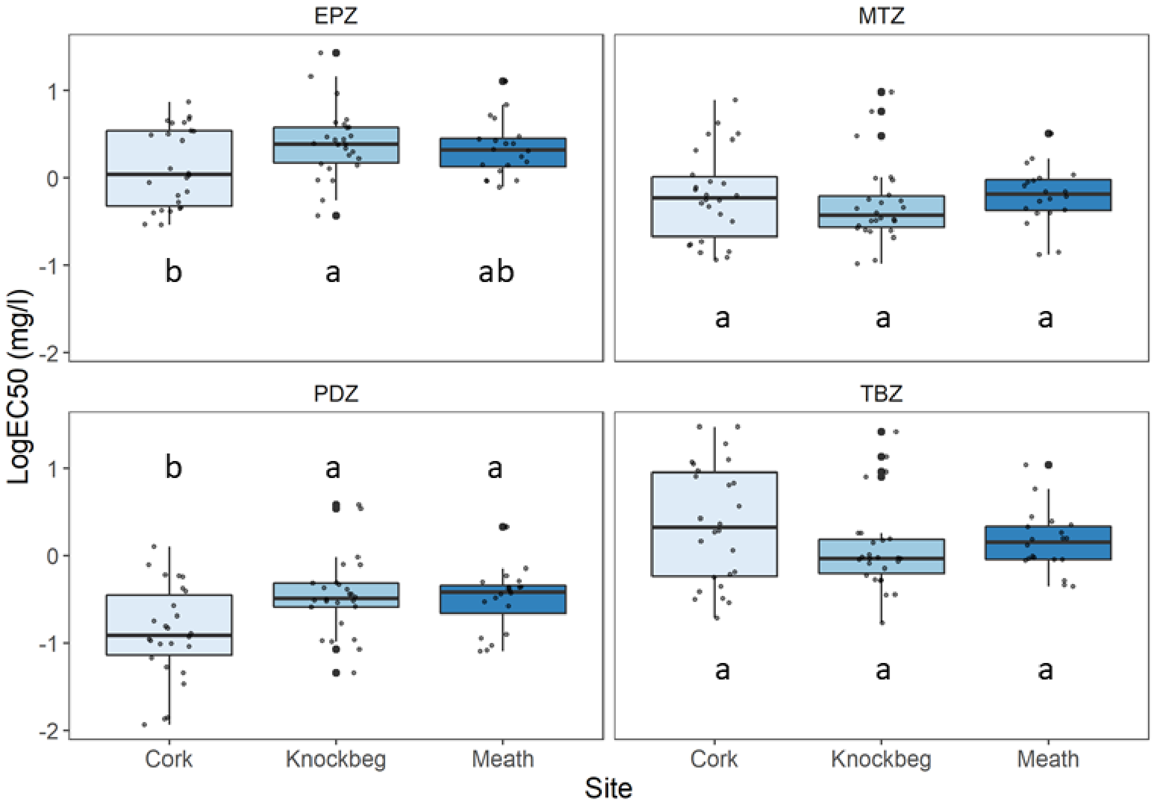

Supplement: Supplementary Image 2 — Differences between the three locations in their sensitivity to the azole fungicides; epoxiconazole (EPZ), metconazole (MTZ), prothioconazole-desthio (PDZ), and tebuconazole (TBZ). Sensitivity presented as LogEC50 (mg l–1), with outliers highlighted in bold. [file Image_2.pdf]
