# Supplementary material for: Development of a PacBio Long-Read Sequencing Assay for High Throughput Detection of Fungicide Resistance in Zymoseptoria tritici
Source: Front Microbiol. 2021 Jun 18;12:692845. doi: 10.3389/fmicb.2021.692845 (PMC8256687; doi:10.3389/fmicb.2021.692845)
Supplement: Supplementary Table 3 — Nucleotide sequences of reference genes in FASTA format. [file Table_3.DOCX]

**Reference sequences for fungicide resistance genes and housekeeping genes of Zymoseptoria tritici**

>cytb (cytochrome b), part of AY247413.1

GCACGTGGGTAGAGGGTTATACTACGGGTCATACAAAGCCCCTAGAACATTAACATGAACAATCGGTACT

ATAATACTAGTTCTGATGATGGCAACCGCATTCTTAGGGTATGTATTACCTTATGGTCAAATGTCTTTAT

GAGGAGCAACAGTTATAACTAACTTATTGAGTGCAATACCTTGAGTTGGACAAGACATAGTTGAATTCGT

ATGAGGTGGATTTTCTGTTAACAATGCAACATTGAACAGATTCTTTGCTCTACATTTCGTTTTACCGTTT

GTGTTAGCTGCATTAGTTTTAATGCATCTAATAGCTTTACACGATACAGCGGGTTCAGGAAATCCTTTAG

GTGTATCAGGTAACTACGATAGATTACCATTCGCCCCTTACTTTATATTCAAAGATTTAATAACAATATT

TTTATTCATTATAGTGTTATCAATATTTATTTTCTTTATGCCTAACGTTTTAGGTGACAGCGAGAATTAT

GTTATGGCTAACCCTATGCAAACTCCACCTG

>CYP51 (14-alpha-demethylase), part of EU418107.2

ATGGGTCTCCTCCAGGAAGTCCTCGCGCAGTTCGACGCGCAATTCGGCCAGACCAGCCTCTGGAAACTT

GTCGGACTTGGATTCCTCGCCTTCAGCACGCTCGCCATCCTCCTCAATGTCCTCTCACAACTTCTCTTCCG

TGGCAAGTTGTCCGATCCGCCACTCGTATTCCACTGGGTGCCCTTCATCGGAAGCACCATCACCTACGG

CATCGACCCATACAAGTTCTTCTTCTCCTGTCGGGAAAAGTATGGAGATGTCTTTACATTCATCCTGCTG

GGAAAGAAGACGACGGTGTGCTTGGGCACCAAGGGCAATGATTTTATTTTGAATGGAAAACTGAAGG

ACGTCAACGCGGAGGAGATATACAGCCCGCTGACCACTCCTGTCTTTGGCAAGGATGTGGTTTATGATT

GTCCCAATTCGAAGCTCATGGAGCAGAAGAAGGTACGGAGAATTGAGAACATACGGGCAAAAGTGCA

GCTATATTGACTTGTCACTTAGTTCGTCAAGTACGGCCTCACAACCTCTGCCCTCCAGTCCTACGTGACC

TTGATCGCCGCCGAGACCCGCCAGTTCTTCGACCGCAACAACCCTCATAAGAAGTTCGCATCGACCAGC

GGCACGATCGATCTCCCACCAGCCCTCGCCGAACTTACGATCTATACTGCCAGCCGATCATTGCAAGGA

AAGGAAGTCCGCGAGGGCTTCGACTCGTCTTTCGCGGACCTCTACCACTACCTCGATATGGGATTCACA

CCGATCAACTTTATGCTTCCGTGGGCGCCCCTTCCCCAGAACCGACGCCGCGATTATGCGCAGAAGAAG

ATGTCCGAGACATACATGTCGATCATTCAGAAGAGACGAGAGTCCAAAACGGGCGAACATGAGGAAG

ACAGTAAGTGAAGTCAGGCACAGGAGGCGTCCAGCTCTCGGAGTCTTGCAGTTGGCCTGTTAGTGGCC

ATGACATCGCCGAGCATTTGCTAACGTCTTGTACCATCAGTGATCCACAACTTGATGCAGTTAGTAAAG

TCACAAGCAGAAGGCGTGCAGATACAGTGTTGCCACGCGTGTCCCACGGCCATGCGGCCCAAGTTCCT

CCCTGGAGACGATGCTGACCCTCGACTTCTAGGTGCAAATACAAGGACGGCAATGCCATTCCCGACAA

GGAGATTGCTCATATGATGATTGCGCTGCTCATGGCCGGCCAGCACTCTTCATCTGCGACCGAGTCCTG

GATCACTCTCCGCCTCGCATCCCGCCCCGACATCCAAGACGAACTCCTCCAAGAACAAAAGGATATGCT

CGGTGTGAACGCCGACGGCAGTATCAAGGAGCTCACATACGCCAACCTCTCGAAACTCACCCTCCTCAA

TCAAGTCGTCAAAGAAACCCTTCGTATTCACGCTCCAATCCACTCCATTCTGCGCAAGGTCAAGTCTCCC

ATGCCCATCGAAGGTACGGCATACGTCATTCCAACCACCCACACTCTTCTGGCCGCTCCGGGCACAACG

AGCCGCATGGACGAGCACTTTCCCGACTGCCTCCATTGGGAGCCGCATCGATGGGACGAGAGCCCGTC

CGAGAAATACAAGCACCTGTCCCCGACGACTGCCCTAGGAAGCATCGCCGAGGAGAAAGAAGACTAT

GGCTACGGCCTGGTAAGCAAGGGCGCGGCGTCGCCATACTTACCCTTTGGTGCGGGACGACACAGATG

TATCGGCGAGCAATTCGCGTACGTGCAATTGCAGACCATTACAGCGACGATGGTTCGCGATTTCAAGTT

TTACAATGTGGATGGCAGCGACAACGTGGTGGGTACGGATTACAGCAGTTTGTTCAGCCGGCCGCTGT

CGCCGGCAGTAGTAAAGTGGGAGAGGAGGGAGGAGAAGGAGGAGAAGAACTGA

>SdhB (succinate dehydrogenase subunit B), part of LT882682.1

ATACCACACAATGGCTCTTCGACTCGCGACGAGGCGCTTTGCGCCAATTGCCTTCCGCCGCGGAATGGCC

ACCACGATCGAGCACACCAAGGAGCCTATCTCCGCCACCGCCGAGGCACTCTCCGCCTCGCGGCCTCCTA

TCAAGGAAACGAAGACGTCGACCGTCAAGGAGCCACAGATGGACGCCGATGCGAAGACAAAGACCTTCCA

CATCTACCGATGGAACCCCGATCAGCCCACCGACAAGCCCCGCATGCAGTCATACACGCTGGACCTGAAC

AAGACGGGTCCTATGATGTTGGATGCTCTGATCAGGATCAAGAACGAGGTGGACCCGACCTTGACATTCC

GAAGGAGTTGCAGAGAGGGTATTTGCGGCAGCTGTGCCATGAACATCGACGGCGTCAACACATTGGCGTG

TTTGTGTGAGTAGTGGACAAAAGATGCATCGGGCCAGTTCGGGAACTGACGATTTGGCAGGCCGCATTCC

CACCGACACAGCAAAGGAGACTCGCATTTACCCACTTCCACACACCTACGTCGTCAAGGACCTCGTGCCA

GACATGACCCAGTTCTACAAGCAGTACAAGTCCATCAAGCCATACCTCCAGCGCGACACTGCACCACCAG

ATGTATGCACCATCCTTCACCTTCCCATACTTGATCTCCTGACTGACACATCCACTTGCAGGGCAAAGAG

AATCGTCAGTCCGTCGCCGATCGCAAGAAGCTTGATGGTCTTTACGAGTGCATTCTCTGCGCATGCTGCA

GCACATCTTGCCCATCCTACTGGTGGAACTCGGAGGAGTACCTCGGACCAGCTGTCCTTCTCCAGTCATA

CCGATGGATCAACGACTCGCGTGACGAGAAGACCGCACAGCGCAAGGACGCACTCAACAACAGCATGAGC

TTGTACCGATGCCACACCATTCTGAATTGCTCAAGGACCTGCCCCAAGGGCTTGAACCCCGCTTTGGCCA

TCGCGGAGATCAAGAAGAGCATGGCTTTCACGGGATAGATGGAACGAGATGATTGGAAAGCGGGGAGTTG

AGGAACGGGATCTGTTTTTGCGTGCTGGCATCGCCTAGATTTGTCCCAGTCAAGAGGGAGCAGTTGTGCC

GATTTTGCGGCATTGTGGCAATCAGTGAGCCACGGTTCTATCGTTTCATGTGTAAATAGCAATTGCATTT

GCGGCTGTCTCGAAATCGACGGAAGAC

>SdhC (succinate dehydrogenase subunit C), part of LT854260.1

GCACTCCCTTGGGTCCTGATGTACCATCTCTCTTCATCCTCCTCCCTCATCTCCACCTACAAGAAAGCCA

AACCCAACGCACTCGCAACACTCAACCCCACAACCGTCCACCCCGTCGTCTGCACCTGCTTATTCGTAAT

CATACTCGCCGTATCCCAAACCAAATGCCTCACTCCATTCAACGAATGAAACGTCACCGGAAGCGCCAAA

ATCGTCTTGGTCAAGACCTGCAACAGCACCGGCCAGGCGCCGAAAGAGGCCGCGAGGGCGGCGGACTCGA

GGTGCCAACCTAGAGATGGCGCGGCGAGGTAGAGGAGTCCGAAGGCGTAGAAGGCTCCCGAGGCGGCGAC

GCCCGTGACGCGGTTGAGAGCGGACAGGTACCAGGTTATTTGCGGTTTGTAGATTGCGAGGTGAGGGGCG

ACGGGGCGGTTGAGGCGTTGTTTGGCGAGGATTTCGTTGCGGGCGTGGGATTCGGAGACGGCGGCTGCTG

TTACTTGGCTGGGAGGGTTGTGAGTGAAGGACGGAGAGAGGAGGAGGTGGGAGGATAGATGGTCTTACCG

GCGCTGCTGCTGGAAGGAGGTGGTGCCGAGGGCGATGGCGGCGGGGGTTGCGAAGCGGAGGGTGGAGGGT

TGGAGGGCTACTGTTGGAGGTCGCAGTGGTTAGTGATTGGGACGATGGTGGAGGGTGGAGGGACGGCAGG

AAGACGTACACCGCCGAAGCGACTGCTGGGTGAGCTTCTGTGCCAACATCGTGAGACGCGGTGTGCCGGA

GCGGAGAGTGGAGGGGTGGTGGATGCGGTGGTGGTGGTGATGTCGACGAAAGAGACAATGCCAGATCAAG

TCTCCAGGATCACAGGACAGGATGATGCCGAGATGTCCCGGTAATAATCGGACCTCTTACTGG

>SdhD (succinate dehydrogenase subunit D), part of LT882679.1

CACTCCTCAAACCGTATCCTCATACACCTCCTACACCTCCTTCATCTTTCGCAACATCCCAGCTTCTCCA

TCTACAACTTCTGCTCAATTTTGTTCTTCGCATCCTTCGCCGTCGCACCCGCCGTCCAAACACGAGCAAT

ACCCGCAGTCAAACCAATATCGTTCGTCTCGAATTCGTACCATCCCCAGCCAACCAGGAAGACAGCAGCG

ACGTTGGCCCAGTCGGCGAGCTTGCGCGTTTTGGGCACGCGCCATGAGGGGAAGTAGTCGGTGATGGCGG

ATCTGGCGAATATGTCAGAATGATACGGGTGGGAAGCTGATGCGCATGTCGTACTGGAAACCAATGTAAG

AGTGGATGATAATCATGCCAATGAAAGTGCCGTCCAAAACCGGGTTGAGCGAGCCAGCAGCGAAGGGGAC

AATAGTCAATGGGATGAGAGCCGCGGAGACGAGTCTGCATATCCTATATCAGCATCCATACCTCATCCTT

CACTTGCGACAACATTTCCCTACCTCTCCATCGTCCAATGGTAGCTTCCATGCGAAGGACTCGGCTCTTT

CACCGGCGCCGGGTCATTGACACCTCCACGGATGACTTGCGGAAGAGGAGGGAGAATAGGTCTCCGCGCA

GTGGTCTGGAAGCCGGAGCGCTGGGTGAATTGAGTGCGGAGGAGGGATGCGGCGGGGAGGGTGGAGGCGC

GCTTGGTGGTGGTTGCGGTGAGGAGCTGGCGGAGAGCAGCGGGGCGGAGGGCGGTGGAGGCCATGGCGAC

GGTGAGGGTGAGTGGAGGGTGGTGTTGTCGATGTGAGTGTAGATGGAAGTGAGGTTGGTTATTCCCGGCT

TGCTTGACGATGATGCC

>act (actin), part of LT854282.1

ACCTCACTCACACCCTCACCACACCGCAAACACCGTCGCAATGGAAGGTAAGCTGCGCCCGCTCCGATT

TTGCCCACTGTTGCCTTTCCTATCCTCAATCGCCTTCATCACTGGCAATGCCTTCATATCGGACACAGGA

GACTGACTCAATTACCTCCCACAGAAGAAGTTGCCGCTTTGGTCATTGACAATGGGTACGGATGCCTTC

TCCCGCCCTCGATTGCGACTCCGCTCAACTCCGACGTCATCCTTCCGCCCACCACACCCCAGTGAAAACA

GCATGGATGAGAGGCGCGAGCGGAGCGCAGCGGACACACATATCCAGTGGACATCACTGACATCCTC

CCAGCTCGGGAATGTGCAAGGCCGGTTTCGCCGGTGACGATGCGCCCCGCGCTGTCTTCCGTGAGTTT

CACACCACTCTATTGACACCTCCAGCGCCAGCCAGCTGACCCTCCCATAGCATCCATTGTCGGCCGACCG

CGCCACCACGGGTATGCGATACCATCTCCTCCTCCTCACCCGACTCGTCTAACACCACCCACAGCATCAT

GATCGGTATGGGCCAAAAGGACTCGTACGTCGGAGA

>BTUB (beta-tubulin-like), part of LT854273.1

CCAGTGCGTAAGTAGCACCAACTCACAATCCTCATTCGACGCGACATGATACTGACAGATCGAATAGGGT

AACCAGGTTAGCCGCCACAATCGAACCCTCCACCTCCCTTCCGCCCACTGACACCTCCACCAGATCGGTG

CTGCTTTCTGGCAGACCATCTCCGGCGAACATGGCCTCGATGGCTCCGGCGTGTACGTAAAAGACACGTG

CACGTGCAAGTGACGGAAACAATGGACAAACATCTGACTCGGCGCAGGTACAATGGCACATCTGACCTCC

AGCTCGAGCGCATGAATGTCTACTTCAACGAGGTACGTGATTTTGGACATTAGCAGCGCCTTCCTACCCC

ACCATGTCGCGTTTACGCCAAGACATTCATCTAACGATGCGCGATTCAGGCCTCCGGCAACAAGTACGTC

CCACGTGCCGTGCTCGTCGATTTGGAGCCGGGTACCATGGACGCTGTCCGTGCCGGTCCTTTCGGTCAGC

TCTTCCGCCCAGACAACTTCGTCTTCGGTCAATCCGGAGCCGGAAACAACTGGGCCAAGGGTCACTACAC

TGAGGGTGCCGAATTGGTCGACCAGGTTCTCGATGTCGTCCGCCGCGAAGCCGAGGGCTGTGACTGCCTC

CAGGGTTTCCAGATCAC

>cal (calmodulin), part of LT854275.1

CAAGGAAGCCTTCTCCCTCTTTGTAAGTTCTACACCGCACCCCTTCTCCTTCACCCCACCTTTCGCAGGG

CTGACCAGCTTTCTCAAACAGGACAAGGACGGCGATGGTATGCTCTCACAGAACTCACATCGAATTCAAT

CCGCGCGACTAACACTGCGCCCAAGGCCAAATCACCACTAAAGAGTTGGGCACCGTCATGCGCTCCCTCG

GCCAGAACCCCAGCGAGTCCGAGCTGCAGGACATGATCAACGAGGTCGACGCCGACAACAACGGCACCAT

CGACTTTCCCGAGTTCCTGACCATGATGGCGCGCAAGATGAAGGACACCGACTCGGAGGAGGAGATCCGC

GAGGCCTTCAAGGTCTTTGACCGCGACAACAACGGTTTCATCTCCTCGGCCGAGCTCCGACACGTCATGA

CCAGCATTGGCGAGAAGTTGACCGACGACGAGGTCGATGAGATGATTCGCGAGGCCGACCAGGACGGTGA

TGGCAGGATTGACTGTAAGAGATGGCTCTGCTGGCATGTTGCGCGATCAGAACACCACTGACACTTTCAC

AGACAACGAGTTCGTCCAATTGATGATGCAGAAGTAGAAAGTCCGCCG

>cyp (cyclophilin), part of XM_003857562.1

CGCACTATGCACAGGAGAGAAGGGCACCGGCAAGTCCGGCTCACCGCTGCACTTCAAGGCCTCTGCCTTC

CATCGCGTCATCAAGCAATTCATGATTCAAGGTGGTGACTTCACAGCTGGCAACGGTACCGGCGGCGAGA

GCATCTACGGCGAGAAATTCGACGACGAGAACTTCCAGCTTAAGCACGAGAAGTCCTTCCTCCTCTCCAT

GGCCAACGCTGGGCCCGGAACCAACGGCAGTCAGTTCTTCGTCACGACTGTCCCCACGCCCCATCTGGAC

GGCAAGCATGTGGTATTCGGCGAGGTCATCAGCGGTAAGAGCGTGGTGCGAGAAGTGGAGAACACACCTG

TTGGACCAAACGACAAACCGGAGAAGGACTGTGTCATCACAGACTGCGGCGAGCTTGCACCAGACGCCAA

CGTCGCGGAGCTCACGAAGAAGCAGAAGGATGCTACCGGTGATGACTACGAAGATTTCCCAGAGGATCAG

CAGAAGGAGGGTGAGGAGTGGAAGGGAACAGAGTTGGCGGACATTGTGGACAATATCAAGTCGTATGGCA

CCGCGGCGTTCAAGGGCACTGATCACGAACTGGCGTTGAGCAAGTACCAGAAAGCTCTGCGATACCTACA

CGAGTACCCGGC

>EF1 (elongation factor 1-alpha), part of LT853695.1

TTGACCGTTCTCAAGCCTCTCGCGCGCACCGAGTTGGCAGCGCAAGAGAAGACAACAAGCAGCTGACAAT

GGATACAGTAAGGAGAAGACTCATATCAACGTCGTCGTTATCGGCCACGTCGACTCCGGCAAGTCGACCA

CCACCGGACGTAAGCGCCTCCTCCCACATTTTGCGACCGATGAGCGACATTCATGCTCACACAATTCACA

GACTTGATCTACAAGTGCGGTGGAATCGACAAGCGTACCATCGAGAAGTTCGAGAAGGTGAGCACTCATT

CCTGGCATCACTGCGGCGACATCACAACGAAAAATTTCTCGCCTCGTCACCCCTCTTGTGGTGGGCGGAG

GGGCAATACTTTGGTGGGGTTTGAGATTTTTGGCGCTTGACCGCTTCTGGTCTCCGCCTTCCATCCTCAG

GCCTCACCACCCATCGCTCCTGCCATCGCAGCAGGCACCACACCACCACATTCCCTTCCATACAATCATG

TCGCATGCAAGTAATGATATGGCCGCTGACAACCATTTCAGGAAGCCGCCGAGCTTGGCAAGGGCTCCTT

CAAGTATGCCTGGGTGCTCGACAAGCTCAAGGCCGAGCGTGAGCGTGGTATCACCATTGATATCGCACTC

TGGAAGTTCGAGACCCCCAAGTACTACG

>GAPDH (glyceraldehyde-3-phosphate dehydrogenase), part of XM_003855615.1

AGGGTCTCATGACCACCATCCACTCCTACACCGCCACCCAGAAGACCGTCGATGGTCCCTCCGGCAAGGA

CTGGCGTGGAGGCCGTACTGCGGCCCAGAACATCATCCCATCCTCCACTGGTGCCGCCAAGGCTGTCGGC

AAGGTCATTCCAGACCTCAACGGAAAGCTCACCGGCATGTCCATGCGTGTGCCAACCGCCAATGTTTCCG

TCGTTGACTTGACCTGCCGCATCGAGAAGGGCGCTACCTACGAGGAGATCACCGCTGCTCTCAAGGAGGC

CTCCGAGGGCGAGCTCAAGGGAATCCTCGCCTACACCGAGGACGACATTGTCTCCAGCGACTTGAACGGC

AACCCTCACTCTTCCATCGTCGATGGCAAGGCCGGCATCTCGCTCAACAAGAACTTCGTCAAGCTCGTCT

CCTGGTACGACAACGAGTGGGGTTACTCTCGCCGTGTCATCGACCTCTTGGCCTACATCGCCAAGGTTGA

CGGCAACGCTTAAGCGAATCGATCCAATATATTTGAGTCTGGTCCGGAAAAGCAAATATTGGGGGAGTCG

AAAAGGACAGCAAGCGAGCGTTGAGTTCAGATACAATGTCTTCGGTCAGAAGCGAATGCGAGTTTGCATC

GGATCAAGCATGGACAAGCGTAG

>hsp80-1 (heat stress protein 80-1), part of LT882679.1

TGAGGATCTCCCACTCAACCTCTCCCGTGAGACCCTCCAGCAGAACAAGATCATGAAGGTGATCAAGAAG

AACATTGTCAAGAAGACTCTCGAGCTCTTCCAGGAGATTGCCGAGGACAAGGAGAACTTTGACAAGTTCT

ACACCGCTTTCGGCAAGAACATCAAGCTCGGTATCCACGAGGATGCTCAGAACCGCCCGGCACTCGCCAA

GCTTCTCCGCTTCAAGTCGACCAAGTCTTCCGACGAGGCCACCTCCCTCCAGGACTACATCACTCGCATG

CCCGAGCACCAGAAGCAAATGTACTACATCACCGGCGAGTCCGACAAGGCCGTCGAGAAGTCGCCATTCC

TTGACGCCCTCAAGAACAAGGGCTTCGAGGTCCTCTTCCTCACCGACCCCATCGATGAGTACGCCTTCAC

CCAGCTCAAGGAGTTTGAGGGCAAGAAGCTCGTCGACATCACCAAGGACTTTGAGCTCGAGGAGACCGAG

GACGAGAAGAAGGCTCGCGAGGCCGAGGAGAAGGAATTCGAATCCCTCGCCAAGTCGCTCAAGAACGTCC

TCGGCGACAAGGTCGAGAAGGTCGTCGTCTCCCACAAGCTCGTCGGCTCCCCTTGCGCCATCCGCACCGG

TCAATTCGGCTGGTCCGCCAACATGGAGCGTATCATG

>PKC (protein kinase C), part of XM_003849459.1

GATCGTGAAAAGGCCATCATTAATGCCGCAAACCAGATGCGGCAGGCAACAAACAATGCCTCTGTCAATT

CTCGCGTCGAATCCAACATTCGCGATGCCAGGCGGAATATTGCATACTTTGAGCAGACGCTACAAGACTT

GAAGGCGCGAAAGATGGGCAATGACATGGGCAATCTTTCCGTGTCTGGGAATGGAGGACCGGCAACACCA

CACCATGGATCCCGAGGAGGCTCTGGCAGCGGCATCAACAACGAGCAAGGCTACGGCGGAGGTGCCGACT

ATGGGAATCCAGGTCCTGGAGGATATAGCATGGGCGGTGCGCCTGGACTCATGCCACCTCGAGCACCATA

CGCGCCAGGTCCTACAGATAGGTCGCCGCGAGCGCGTCCGAACTATAGCAAGCTCGATCTGATCAAATAC

GACACCGCTCACCTCGGCCCACGAATCCAGCTCATGTTGTCCCAGCTCGAATTCAAACTTTCCGTGGAGA

AGCAGTACAAGGACGGCATTGAAAAGATGGTCACACTATACCAAATGGCAGGCGATCGTAAGAGCAGAGG

CGAGGCAGAACTGCGCCGAATGGAGAGCAATCAAAAGATCCAGCTTCTTACGAGGGCACTGCGGAGATAT

GAAGACCTTCACGTTGATGTTGAGAATGGCGCAGATGGCGCGGATGACGATAGTCTGGACACTCCCAGCC

>TFC1 (transcription factor class C), part of LT854280.1

GAACGTAGGTGCTGCAAACATTCCTTCAACAGACAACATCTTACGGAGAACAGGCTCTCGAACATCATG

TAGGTGACTCAAGACTCAGCAGCAGACAGAATGCGCTTCCAGAACCGGTCGTAGGCGTTTCCCTTCGG

CCCAATGACCCATTGGCGAAGAAGATTGCGTCGACGGGCATCGAGACTCGGAACGTTCTGTTGAAAGT

TACAGTGCCGAAGCAAACAGGTCGCAAGCGCAAAAGGGGCTCCGATGAGCCCTTCACTGAGCCAGCTC

AGCCCGTGCGGGTGTGCAGCAACCCCAAGGCTGCTCAGATTGTGCGAAGACTTCGCGACAATATGGAC

AGATATAGTGTCGAGGCTGTAGGAATGGTGCGCGAGACACACAGATTCCGCACGCTGCCAGACTTTCA

GCTTCGCAACGCCGACCTGCCCATCATGAGAGAGATTCGAGATCACCTCATGGTATCGGACTACGACA

GATTGAAGAGCTTCCATGTCGACCTCGGATCTAATCCTTCGATGGCGACCGCATTCCCCGGACCTCCGG

CGTTCACCAGCATGGACATGCCCTACAAGTATGACTACGAACAGGCCGCTGGTGTTGTCTTCGAGAAA

GACGAGACCGGCAATTTGACCTCCAAAAACCTGTCAGCACCACC
